# A Case of Fibrous Pseudotumor in the Scrotum: Challenge for Diagnosis and Testicular Preservation

**DOI:** 10.1155/2018/6904827

**Published:** 2018-01-17

**Authors:** Hirotake Kodama, Shingo Hatakeyama, Teppei Matsumoto, Toshikazu Tanaka, Hirotaka Horiguchi, Yuka Kubota, Hayato Yamamoto, Atsushi Imai, Takahiro Yoneyama, Yasuhiro Hashimoto, Takuya Koie, Chikara Ohyama

**Affiliations:** ^1^Department of Urology, Hirosaki University Graduate School of Medicine, Hirosaki, Japan; ^2^Department of Advanced Transplant and Regenerative Medicine, Hirosaki University Graduate School of Medicine, Hirosaki, Japan

## Abstract

A paratesticular fibrous pseudotumor is a relatively rare benign disease. Preoperatively diagnosing a fibrous pseudotumor is challenging because distinguishing these masses from malignant tumors on the basis of clinical and radiological findings can be difficult. We present a case of a 28-year-old man who presented with a painless palpable mass in the right scrotum; the fibrous pseudotumor of the tunica vaginalis was treated with organ-sparing surgery. Computed tomography and magnetic resonance imaging revealed paratesticular tumors. Testicular tumor marker levels were within normal limits. We scheduled the patient to undergo tumor biopsy combined with intraoperative rapid diagnosis. Frozen section assessment suggested a fibrous pseudotumor without malignancy. We successfully performed organ-sparing surgery. Testicular-sparing surgery combined with frozen section assessment is primarily used for treating paratesticular fibrous pseudotumors.

## 1. Introduction

Intrascrotal lesions, usually hydrocele testis, are not rare in the male population. While 95% of testicular lesions are malignant, most paratesticular lesions are benign. Fibrous pseudotumor is the second most common intrascrotal lesion after adenomatoid tumors [1]. Fibrous pseudotumors comprise only 6% of paratesticular lesions and are observed at any age, with the incidence peaks between the ages of 20 and 40 years [2]. Because these tumors are benign, testis-sparing surgery, rather than radical orchiectomy, should be essentially performed to preserve fertility in these patients. However, the preoperative diagnosis of a fibrous pseudotumor is challenging. Distinguishing these tumors from malignant lesions on the basis of clinical and radiological findings can be difficult owing to the lack of a typical signal and tumor size. Orchiectomy for testicular fibrous pseudotumors has been previously reported [1, 3–7]. Here, we present a case of fibrous pseudotumor of tunica vaginalis which was treated with testicular-sparing surgery.

## 2. Case Presentation

**Figure 1:**
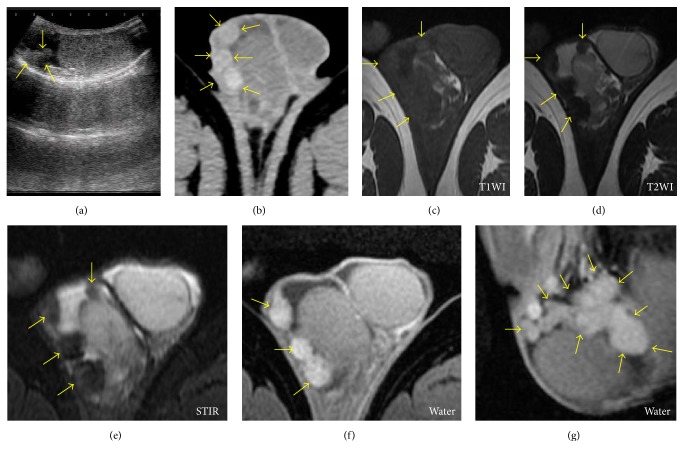
*Imaging of the fibrous pseudotumor in the scrotum*. Scrotal ultrasound revealed a normal testicle and multiple 3 to 7 mm hyperechoic lesions adjacent to the right testis ((a), arrows). Contrast-enhanced computed tomography (CT) showed high-density paratesticular tumors ((b), arrows). Magnetic resonance imaging (MRI) showed iso- and low-intensity paratesticular tumors in T1- ((c), arrows) and T2-weighted MR images ((d), arrows), respectively. Short TI inversion recovery (STIR) MRI showed low-intensity paratesticular tumors ((e), arrows). Water MRI showed high-intensity tumors ((f) and (g), arrows). T1WI: T1-weighted image; T2WI: T2-weighted image.

**Figure 2:**
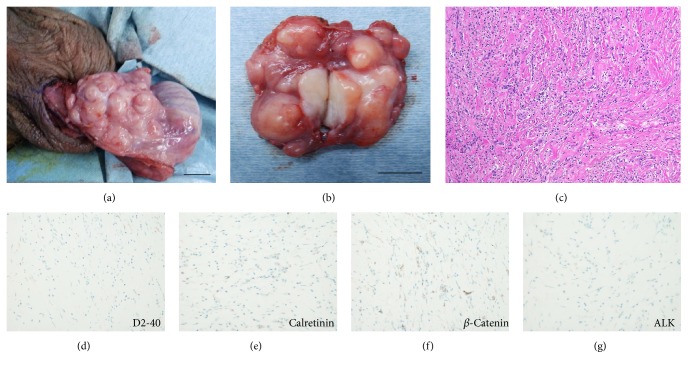
*Macro- and microscopic findings of the fibrous pseudotumor*. Macroscopic findings showed many paratesticular white pedicle masses (a). We excised the paratesticular white masses (b) and evaluated them via frozen section (scale bar = 1 cm). After it was determined that the masses were not malignant, the tunica vaginalis was excised and they were totally removed. Pathological findings showed the proliferation of typical fibroblasts distributed in multidirectional bundles of dissociated collagen fibers (c). Immunohistochemical staining was negative for D2-40 (mesothelioma, (d)), calretinin (mesothelioma, (e)), *β*-catenin (desmoid-type fibromatosis, (f)), and anaplastic lymphoma kinase (ALK and inflammatory myofibroblastic tumor, (g)).

A 28-year-old man presented with a painless palpable mass in the right scrotum. Scrotal ultrasound revealed a normal testicle and multiple 3 to 7 mm hyperechoic lesions adjacent to the right testis (Figure 1(a)). Contrast-enhanced computed tomography (CT) revealed high-density paratesticular tumors (Figure 1(b)). The surface coil magnetic resonance imaging (MRI) with a 1.5 tesla scanner (GE Healthcare Signa HDxt 1.5T) revealed iso- and low-intensity paratesticular tumors on T1- (Figure 1(c)) and T2-weighted MR images (Figure 1(d)), respectively. Short TI inversion recovery MRI showed low-intensity paratesticular tumors (Figure 1(e)). Water MRI showed high-intensity tumors (Figures 1(f) and 1(g)). The levels of testicular tumor markers such as *α*-fetoprotein, *β*-human chorionic gonadotropin, and lactate dehydrogenase were normal. We planned tumor biopsy combined with intraoperative rapid diagnosis. Scrotal incision was performed to explore the scrotum, and paratesticular white pedicle masses were observed (Figure 2(a)). Over 20 nodules were observed at the tunica vaginalis. The maximum size of nodule was approximately 15 mm. Frozen section assessment was performed, and intraoperative rapid diagnosis suggested a fibrous pseudotumor without malignancy. We excised the paratesticular white masses (Figure 2(b)) and successfully performed testicular-sparing surgery. Pathological findings revealed the proliferation of typical fibroblasts that were distributed in multidirectional bundles of dissociated collagen fibers (Figure 2(c)). Lymphocyte infiltration including immunoglobulin G4- (IgG4-) positive plasma cells was observed. Tissue IgG4 counts and IgG4/IgG ratios were 10 positive cells per high-power field on average and 10%, respectively. Ki-67 labeling index was 2%. Immunohistochemical staining was negative for D2-40 (mesothelioma, Figure 2(d)), calretinin (mesothelioma, Figure 2(e)), *β*-catenin (desmoid-type fibromatosis, Figure 2(f)), and anaplastic lymphoma kinase (ALK) (inflammatory myofibroblastic tumor, Figure 2(g)). No recurrences have been noted after 12 months of follow-up.

## 3. Discussion

A paratesticular fibrous pseudotumor is a relatively rare benign disease and was first reported in 1904 by Balloch [1]. Pseudotumor was coined by Mostofi and Price in the AFIP Atlas 1973 [8]. The overall incidence of paratesticular fibrous pseudotumor is exceptionally rare with approximately 200 cases reported to date [3, 9–16]. The relative incidence of paratesticular fibrous pseudotumor in relation to testicular germ cell tumors was reported to be 1 : 200 [9]. Approximately 85% of cases originate from the tunica vaginalis, followed by the epididymis, spermatic cord, or tunica albuginea. Although the etiology is unknown, a history of trauma, surgery, infection, and inflammatory hydrocele is suggested to be associated with the development of a disease. Recently, it is suggested that paratesticular fibrous pseudotumor might be a part of IgG4-related sclerosing disease, which has an abundance of IgG4-staining plasma cells [9, 12, 17]. The diagnosis of IgG4-related disease is based on the combined presence of the characteristic histopathological appearance and increased numbers of IgG4^+^ plasma cells. The critical histopathological features are a dense lymphoplasmacytic infiltrate, a storiform pattern of fibrosis, obliterative phlebitis, tissue IgG4 counts, and IgG4/IgG ratios (approximately >40%) [18]. In addition, IgG4 elevation in serum may support the diagnosis. However, the proportion of IgG4-positive cells among IgG-producing plasma cells in our case was only 10%. Unfortunately, immunoglobulin serum profiles had not been taken in our patients. However, similar to our case, no etiological factor can be detected in most cases. Because the pathogenetic relevance of this test had not been considered during the clinical management, further studies are necessary to clarify the etiology of paratesticular fibrous pseudotumor.

Macroscopically, these nodules have typical characteristics such as single presentation or multiple presentations, are firm with a white tan, are whorled cut surface, and originate from the testicular tunics or epididymis [3]. Despite their typical tumor appearance, the preoperative diagnosis of a fibrous pseudotumor is challenging. Several authors have reported the ultrasonographic appearance of this entity [13, 19–22]; however, the findings on ultrasound are frequently nonspecific. They may present as either hyperechoic or hypoechoic lesions depending on the degree of calcification, hyalinized collagen, and granulation tissue [10]. Therefore, it is difficult to distinguish paratesticular fibrous pseudotumor from others by ultrasound findings alone. A recent study on ultrasonographic appearance of the tumors suggested the utility of constant acoustic shadowing of these lesions. Ohana et al. suggest that the presence of multiple and confluent extratesticular masses associated with significant acoustic shadowing and low-to-absent Doppler signal leads to the diagnosis of pseudotumor of the tunica vaginalis. [14]. Although case reports with MRI are limited, some studies have reported a specific appearance with an extratesticular multiple nodular lesion that exhibits intermediate-to-low signal intensity on T1- and T2-weighted images, with little or no gadolinium enhancement [20, 23]. Our result showed similar observation and suggested that water imaging might be useful for tumor visualization (Figures 1(f) and 1(g)). However, distinguishing these masses from malignant tumors on the basis of clinical and radiological findings can be difficult.

Depending on the difficulty of diagnostic imaging, orchiectomy for testicular fibrous pseudotumors has been reported in several case reports [1, 3–7]. Because benign tumors are frequently reported for testicular and paratesticular tumors (*n* = 36/43, 83.7%) [5], testicular-sparing surgery combined with frozen section assessment is optimal when fibrous pseudotumor is suspected. However, frozen section assessment for testicular and paratesticular lesions has not been well utilized because clinical diagnosis is typically accurate [5]. Not many studies have suggested that frozen section assessment is accurate and effective for preventing radical orchiectomy [5, 24, 25]; however, a recent study suggested the efficacy of frozen section assessment for testicular and paratesticular lesions for which malignancy is suspected. Their results suggested that frozen section assessment aided in preventing unnecessary radical orchiectomy (83.7%) for a benign diagnosis [5].

## 4. Conclusion

Testicular-sparing surgery combined with frozen section assessment is primarily used for treating paratesticular masses.
